# The potential impact of singing on young children’s health and well-being: a longitudinal perspective

**DOI:** 10.3389/fpsyg.2026.1793612

**Published:** 2026-03-18

**Authors:** Graham Frederick Welch, Hazel Baxter

**Affiliations:** Institute of Education, University College London, London, United Kingdom

**Keywords:** children, development, health and well-being, longitudinal study, singing

## Abstract

The article reports data from an ongoing research evaluation of the impact of a special singing programme with young children in a London Primary school. A particular focus is on the extent to which any wider benefits of singing are evidenced in terms of participant children’s health and well-being. The research data were collected from children aged six to eight across two school academic years. The programme is being led by professional singers from a charitable singing foundation who make regular visits to the school to work with children, their teachers and teaching assistants. Children’s singing behaviour and development was assessed by combining data from the Singing Voice Development Measure (SVDM) and a revised model of vocal pitch-matching development (VPDM). Children’s perception of their health and wellbeing was assessed through the Very Short Wellbeing Questionnaire for Children (VSWQ-C) and the PANAS-C measure of emotional wellbeing (modified for younger children). Longitudinal data analyses from four separate data collections over 18 months suggest that children’s singing competency continued to improve over time, with younger children showing greater progress due to their less developed skills initially. Participants outperformed national averages in singing competency for children of equivalent ages. Children consistently self-reported high well-being ratings, with a reduced variability in negative responses, particularly among younger children. The data analyses suggest that the programme supported children’s singing development. Although there is no direct statistical evidence linking singing with health and well-being, the findings align with global research highlighting the mental, physical, and social benefits of singing. We speculate that the programme continues to contribute positively to the school’s culture and, by implication, potentially serving as a protective measure for their health and well-being. Ongoing research needs to explore this possibility.

## Background

The UK Government’s official policy on pupils’ mental health and well-being suggests that they see a clear and positive link with successful development and achievement (DFE, 2025). Within a global context, an example is provided by the world’s largest school-based mental health initiative programme which has been based in Chile since 1998 as part of their ‘Skills for Life’ (‘*Habilidades para la Vida’*) programme. Longitudinal analyses in 2016 of data from N = 37,397 pupils across their first three school Grades (ages 6–9 years) suggested that mental health in Grade one (aged 6–7y) was a significant predictor of subsequent academic outcomes in Grade three (aged 8–9y; Murphy et al., 2015). Subsequently, a revised version of the original Elementary school programme for ‘at risk’ pupils was implemented in over 700 of Chile’s Middle schools and with positive results concerning significantly improved school attendance and social relationships (Canenguez et al., 2023).

Here in the United Kingdom, the concern for pupils’ mental health and well-being in schools continues to be a priority, particularly following the negative impact of Covid-19. The pandemic severely disrupted schooling and was reported to foster a sense of isolation and worry, particularly related to dangers to physical health and the need for quarantine. For example, an internationally-focused systematic review of *N* = 113 studies on the impact of the pandemic on children and young people in North America, China and Europe reported widespread increases in fear, anxiety, depression, loneliness and behavioural issues (Naff et al., 2022). These outcomes were also noted as more prevalent for children in lower-income families (Ravens-Sieberer et al., 2021). Nevertheless, among the wealth of studies were reports of children’s and young people’s resilience—a protective factor—in the face of such health challenges. Positive strategies included exercise, the use of technology, and engaging in creative outlets, such as listening to popular music—as exampled in China (Xian et al., 2024) and Spain (Martínez-Castilla et al., 2021), and making music—as exampled in Canadian adults (Barbeau et al., 2024), in Scottish children (Robb et al., 2023), and for many young people across the United Kingdom (Youth Music, 2022). The results of an online survey of 5,619 adults across 11 countries indicated that musical experiences were the most effective activities during Covid-19 for reducing negative emotions and fostering a sense of social connection (Granot et al., 2021).

Within this literature on the positive impacts of music on health and well-being are studies related to singing. For example, a large-scale German study recorded the effects of choir singing on adult mental health, with larger improvements in mental health being related to longer choir membership, more singing hours per week, and personally having a high engagement with choral activity (Robens et al., 2024). Positive musical activity, including singing, influences neurochemical production, including dopamine, serotonin and oxytocin, thus helping to modify internal perceptions of stress and likely to increase a sense of social inclusion (Chanda and Levitin, 2013; Kreutz et al., 2004; see Levitin, 2024). Furthermore, neuroscientific studies suggest that singing should be considered as a whole brain activity because of the number of different neural regions that are networked across the brain’s two hemispheres in musical activities which embrace the voice and language (lyrics) (e.g., Kleber and Zarate, 2019; Särkämö and Sihvonen, 2018; Sihvonen et al., 2024). The power of singing has also been demonstrated in enabling positive communicative and psychosocial outcomes in post-stroke aphasia in adults (Tamplin et al., 2013; Siponkoski et al., 2023), as well as in children (Thompson and Schlaug, 2015) and also in a multi-modal singing-based interventional with refugee children (Mohammadhosseini and Schmid, 2025).

Although there is less research on the wider benefits of successful singing for children’s health and well-being (e.g., Schmid, 2025), we can hypothesise that impacts may be similar to those reported for adult human populations. These include support for the immune system because singing is an aerobic activity which increases oxygen intake. Singing also modifies the release of stress hormones and increases oxytocin, supports cardiovascular function, improves mental alertness and—in choral groups—allows joint breathing cycles to match phrases in the music (see Welch et al., 2019, for an overview, including chapters by Theorell (2019) and Clift and Gilbert (2019)). Regarding child populations, a London-based study with *N* = 60 children aged 7–11 years from two Primary schools found that overall psychological well-being data correlated strongly with children’s identities as singers (Hinshaw et al., 2015). In New Zealand, a daily ‘singing for well-being’ action research project, initiated in a Primary school following the 2010–2011 earthquakes, reported positive benefits on children’s emotions, moods, and sense of collective identity (Rickson et al., 2018). In England, a recent study reported the positive impact of daily singing for 2 weeks on the subjective well-being of a class of *N* = 27 8–9-year-olds (Davies et al., 2023). In Sweden, drawing on the findings from existing literature, there is an ongoing major project ‘Singing, health and well-being in school—a societal matter’ (‘*Sånghälsa i skolan—en samhällelig angelägenhet’*) in which research has focused initially on how to enable Primary school teachers to support children’s singing on a daily basis (Horwitz et al., 2024).

A systematic review of the effects of group singing on the well-being and psychosocial outcomes of children and young people (Glew et al., 2021) found 13 studies which fitted their inclusion criteria. While having reservations about the wide variations in researchers’ methodological approaches and an apparent lack of systematic controls, the authors nevertheless suggest that ‘social connectedness is a potential mechanism by which group singing impacts well-being’ (p. 257). This hypothesis is supported elsewhere in (a) an extensive inter-disciplinary proposal for the biological and cultural evolution of human musicality (Savage et al., 2021), (b) the neurological data related to links between the release of oxytocin and a sense of social bonding (Kreutz et al., 2004; Theorell, 2019), and (c) in a major national study of singing and social inclusion in *N* = 6,087 children (Welch, 2014).^1^

## Research context

The VOCES8 Foundation is a London-based charity which is recognised as a leading provider of world-class performances as well as singing learning and participation projects in the United Kingdom, France and United States. As part of the Foundation’s community engagement and enrichment programme in London, members of the team have been offering specialist singing input to children and teachers in a London Primary school. This programme began in the academic year 2022–2023 and continues into 2025–2026. As part of the programme, the Foundation requested an independent longitudinal evaluation of two classes of participant children’s singing behaviours and development in both the 2023–2024 (beginning in January 2024) and 2024–2025 academic years, including whether there was any evidence of a wider impact of the VOCES8 singing programme on the children’s health and well-being. The research team were invited to undertake the independent evaluation on the basis of having a recognised international expertise and publication in the nature of children’s singing behaviour and development, as well as in the professional music education development of generalist Primary teachers.

The focus Primary school is in East London, north of the River Thames. Based on the English Indices of Deprivation (2019), nationally, the local area around the school is ranked in the top 30% of greatest deprivation nationally and has 26.6% of children living in income deprived households, being nationally ranked as 14th in the top 20 most deprived local authority districts. The local area also has the highest proportion nationally of older people (43.9%) in income deprived households. It is the worst local area in London for income deprivation overall (2024 data). Of the 205 children in the school in 2024, 61% (125 pupils) have some form of special educational need and one third (32.5%) of children are eligible for free school meals—seen as one of the main indicators of childhood deprivation.

The school’s most recent Ofsted (Office for Standards in Education, Children’s Services and Skills) inspection in February 2024 rated the quality of education as continuing to be ‘outstanding’, the same rating as awarded in 2012. The inspection report states that this is ‘an exceptional school where pupils are nurtured and supported to achieve their very best in all areas of school life’ (p2). Although there is no specific mention of music (nor the arts) in the inspection report, it is evident from our evaluation visits that music is often a common collective activity for children in the school hall, such as for school assemblies.

The latest pupil attainment data for 2024–2025 is in line with national data in terms of the Early Years Foundation Stage profile (age 5y) and the Year 1 phonics assessments (aged 6 years). For the older children, Key Stage 2 assessments (age 10+) are in line with or above national data and with many children achieving well. This past Summer (2025) the school appointed a new Headteacher, following the retirement of the previous successful incumbent.

The whole-class singing programme was led by professional singers from the VOCES8 Foundation who visited the school approximately every 2 weeks across 2 six-monthly periods (January to June 2024 and January to June 2025). Class teachers were expected to lead collective singing with their classes between such visits and the programme concluded each Summer with a performance for parents in central London at the VOCES8 Centre. The Foundation’s singing programme draws on its in-house method^2^ which includes sequential repetition that links voice and gesture, as well as a range of age-appropriate repertoire.

## Longitudinal findings from an evaluation of the VOCES8 primary school singing programme across two school years (2024–2025)

The current report is a continuation of our 2024 empirically based investigation into the potential well-being benefits of a VOCES8 Foundation’s singing programme with two classes of young children in the East London Primary school (Welch and Baxter, 2025). The original study focused on possible changes in key measures of singing and well-being across a six-month period. This has now been extended in the current article by research across a second academic year, offering 18 months in total of longitudinal comparison in pupils’ responses. The earlier evaluation reported (i) evidence of a significant improvement in children’s singing competency during the year, which was comparable favourably with national singing data, and (ii) that the children’s perceptions of their health and well-being were sustained across the research period (January to June 2024). Although there was no clear evidence statistically of any direct link between singing, health and well-being, primarily this related to a relative ‘ceiling effect’ in the psychological data where children’s responses were clustered towards the maximum. (A ceiling effect is observed when a large number of participants achieve a maximum or near-maximum score on a given test.) Notwithstanding some individual but not consistent variation, the children were very positive about their health and well-being throughout the research period. It was noted that the older children in the programme (aged 7–8 years) were likely to have been subject to Covid-19-related restrictions during their nursery years when, although there was some limited opening of Primary schools, non-compulsory pre-school provision (aged 3–5y) was closed.

In this second year of data collection, the same two research tools were used to investigate any changes over time in the focus singing, health and well-being measures (see below).

## Research tools

Following our earlier 2024 report, the findings draw on two main strands of quantitative research at the school.^3^ These relate to (i) children’s singing behaviour and development, and (ii) their perceived health and well-being. With regards to (i) their singing behaviour and development, each child was assessed individually against their performance of two well-known songs—*Twinkle, Twinkle Little Star* and *Happy Birthday*—using two established rating scales (Rutkowski, 1997; Welch, 1998—see Mang, 2006; Welch et al., 2012 for more detail). The resultant data from the four ratings (two per song) was combined into a ‘normalised singing score’ (NSS) out of 100 for each child. This enables ease of comparison between children and offers evidence of any changes in their singing competency over time. In general, score ratings above 90 out of 100 suggest an overall tendency for the child’s singing to be in-tune and in time, with accurate lyrics and an appropriate singing register usage. Vocal behaviours rated as scoring 40 or below imply that the child is essentially still at a developmental phase of speaking the lyrics, with little sense of musical key or melodic shape. All individual singing behaviour scores were agreed by the two members of the research team, drawing on a visual display of the child’s singing behaviour using the software programme ‘SING&SEE’^4^ running on a MacBook Pro.

The assessment of (b) children’s perceived health and well-being was based on their responses to a set of simple statements presented on a tablet computer screen, drawing on previous research by Smees et al. (2020). Smees et al. (2020) had designed and validated two associated measures: the *Very Brief Well-Being Questionnaire for Children (VSWQ-C),* a health-related quality-of-life scale designed to be suitable for young children from the age of 6 years. Their research also adapted an existing measure of perceived emotional experiences, the *Definitional Positive and Negative Affect Schedule for Children (dPANAS-C)* which consists of 10 statements about feelings, five of which are positive and five are negative. Care was taken to ensure that the balanced mix of negative and positive statements were understood by each participant in order to mitigate any potential tendency for children to seek to please the adult research team by providing what they perceived to be the ‘correct’ answer.

## Longitudinal findings: (1) singing behaviour and development

**Figure 1:**
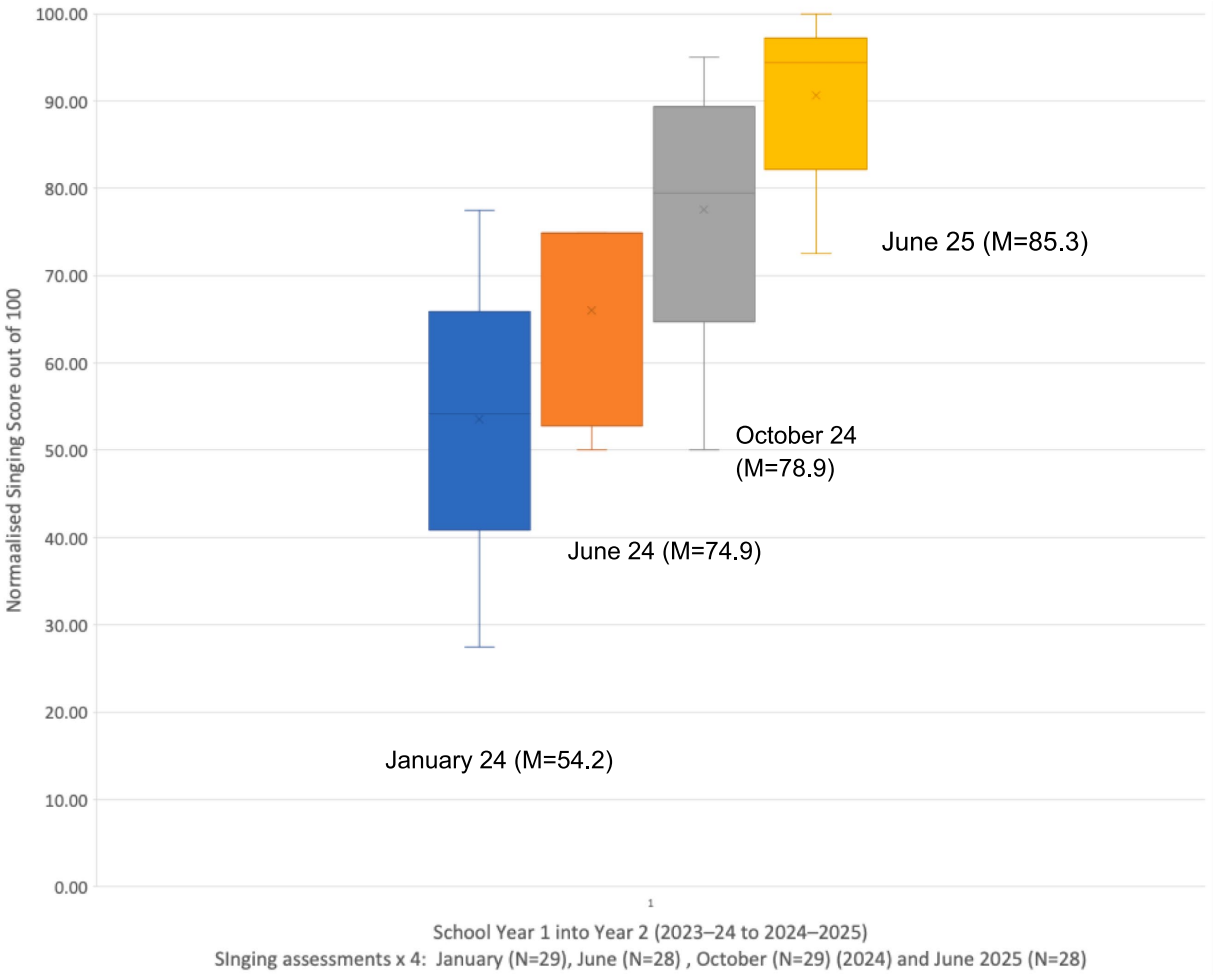
Longitudinal singing data for Year 1 into Year 2 (January, June, October 2024, and June 2025).

**Figure 2:**
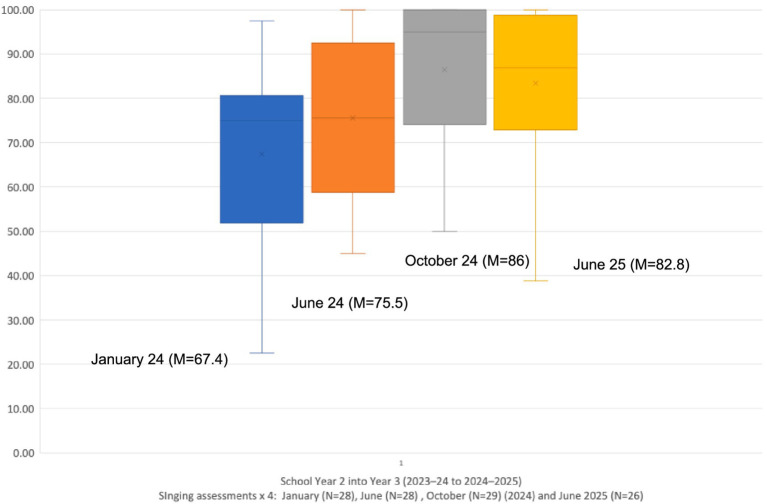
Longitudinal singing data for Year 2 into Year 3 (January, June, October 2024, and June 2025).

The participants were children in school Years 1 and 2 in 2023–2024 (ages 6–7y), moving up to Years 2 and 3 (ages 7-8y) respectively in the academic year 2024–2025. Each child’s singing competency was assessed four times, namely at the research baseline in January 2024, again at the follow-up in June 2024 at the end of the initial year of the VOCES8 programme, and then again in October 2024 at the beginning of the programme’s second year and finally in June 2025. The Normalised Singing Scores (NSS) for each Year group of children were collated and are illustrated as histograms and means in Figures 1, 2 for the four time points (January, June and October 2024, and June 2025).

As can be seen from these two Figures, there is a clear improvement in mean singing competency across the four time points for each class, as measured in their individual sung performance of the two target songs (*Twinkle, Twinkle* and *Happy Birthday*). Applying a Friedman Test for Repeated Measures reveals that there was a highly statistically significant improvement in the singing of the Year 1 children across 2024 (January to June) and into Year 2 [October 2024 and June 2025; *X^2^_r_* = 44.59 (3, *N* = 32), *p* < 0.01].

Similarly, using the same statistical protocol, a Friedman Test for Repeated Measures, the singing of Year 2 children also shows a statistically significant improvement as they progressed into and across Year 3 [*X^2^_r_* = 27.24 (3, N = 32), p < 0.01].

The children’s singing data are also shown in Table 1 with a comparison to children of the same age (school Years 2 and 3) from our national NSS dataset drawn from our *Sing Up* programme evaluation (2007–2012; Welch et al., 2012; Papageorgi et al., 2022).

**Table 1:** Longitudinal mean normalised singing scores (NSS) for participant children at four evaluation points (2024–2025) compared with NSS data for children of the same age from the national dataset.

Mean normalised singing scores (NSS)	Baseline Jan 2024	Follow up June 2024	Follow up October 2024	Follow up June 2025	National dataset			
					Mean non-sing up scores (NSS) Yr 2 (*N* = 15)	Mean sing up (NSS) Yr 2 (*N* = 957)	Mean non-sing up scores (NSS) Yr 3 (*N* = 906)	Mean sing up (NSS) Yr 3 (*N* = 1,498)
Year 1 to Year 2	54.2	74.9	79.5	85.3	60.9	72.1	–	–
Year 2 to Year 3	67.4	75.5	86.0	82.8	–	–	66.4	73.9

The maximum normalised singing score (NSS) is 100.

**Figure 3:**
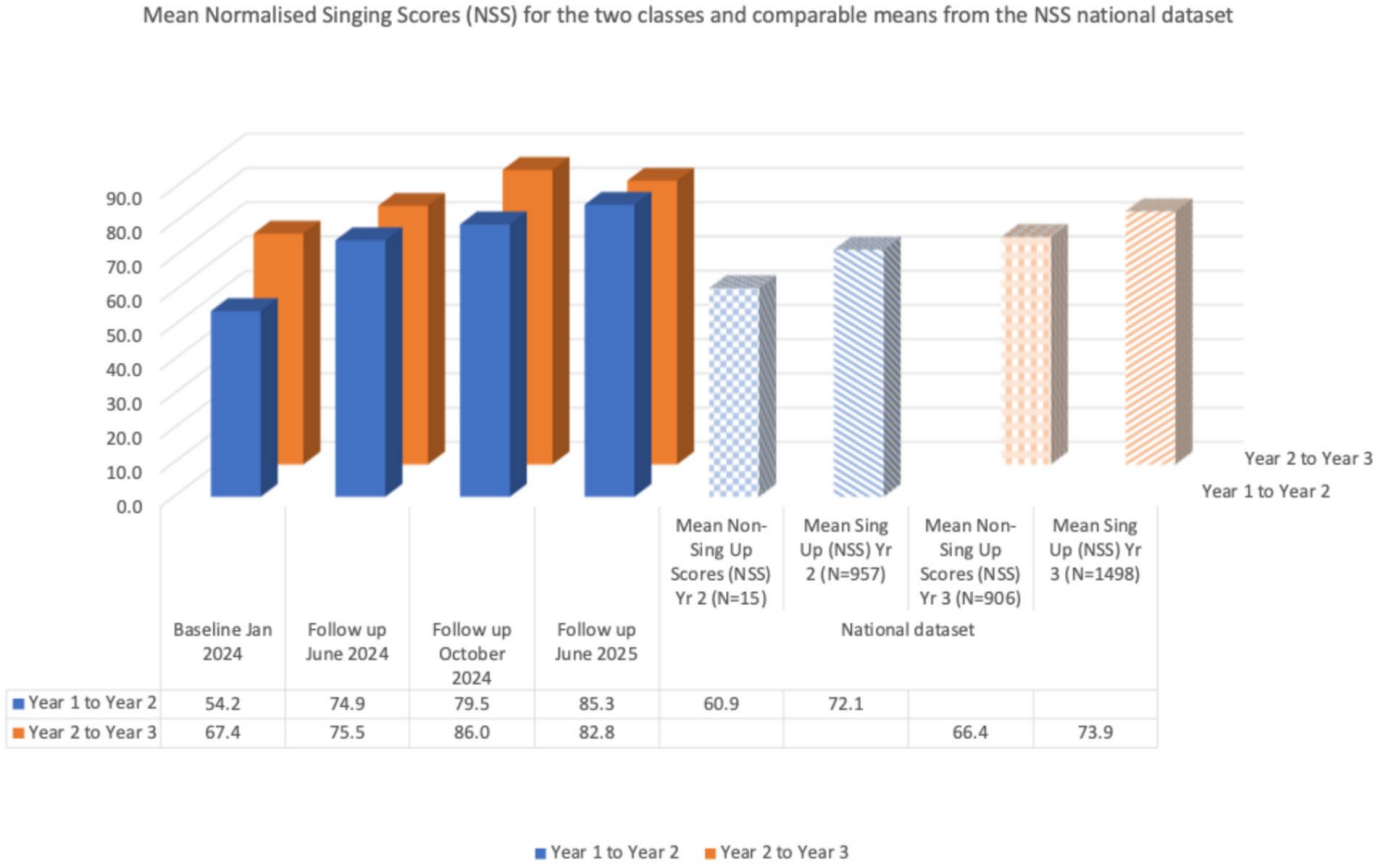
A visual representation of the data in Table 1 to show the longitudinal trends over time for the participant children, plus a comparison with the national data for children of the same age (*N* = 972 children in Year 2; *N* = 2,404 children in Year 3).

These data show both an improvement for each class group in their mean singing competency over time, and also that their mean singing competency compares favourably with the means in the national dataset for children both inside and outside the Sing Up programme at the time of assessment (also illustrated in Figure 3).

## Longitudinal findings: (2) health and well-being

### Very brief well-being questionnaire for children

With regards the data on health and well-being, these are reported separately below for the two scales that were used in the assessment. Firstly, the *Very Brief Well-Being Questionnaire for Children (VSWQ-C)* data derives from children’s responses to a four-item self-report questionnaire which covers self-perceptions of key aspects of their lives: home life, school life, friends, and health (*cf.* Smees et al., 2020).

**Figure 4:**
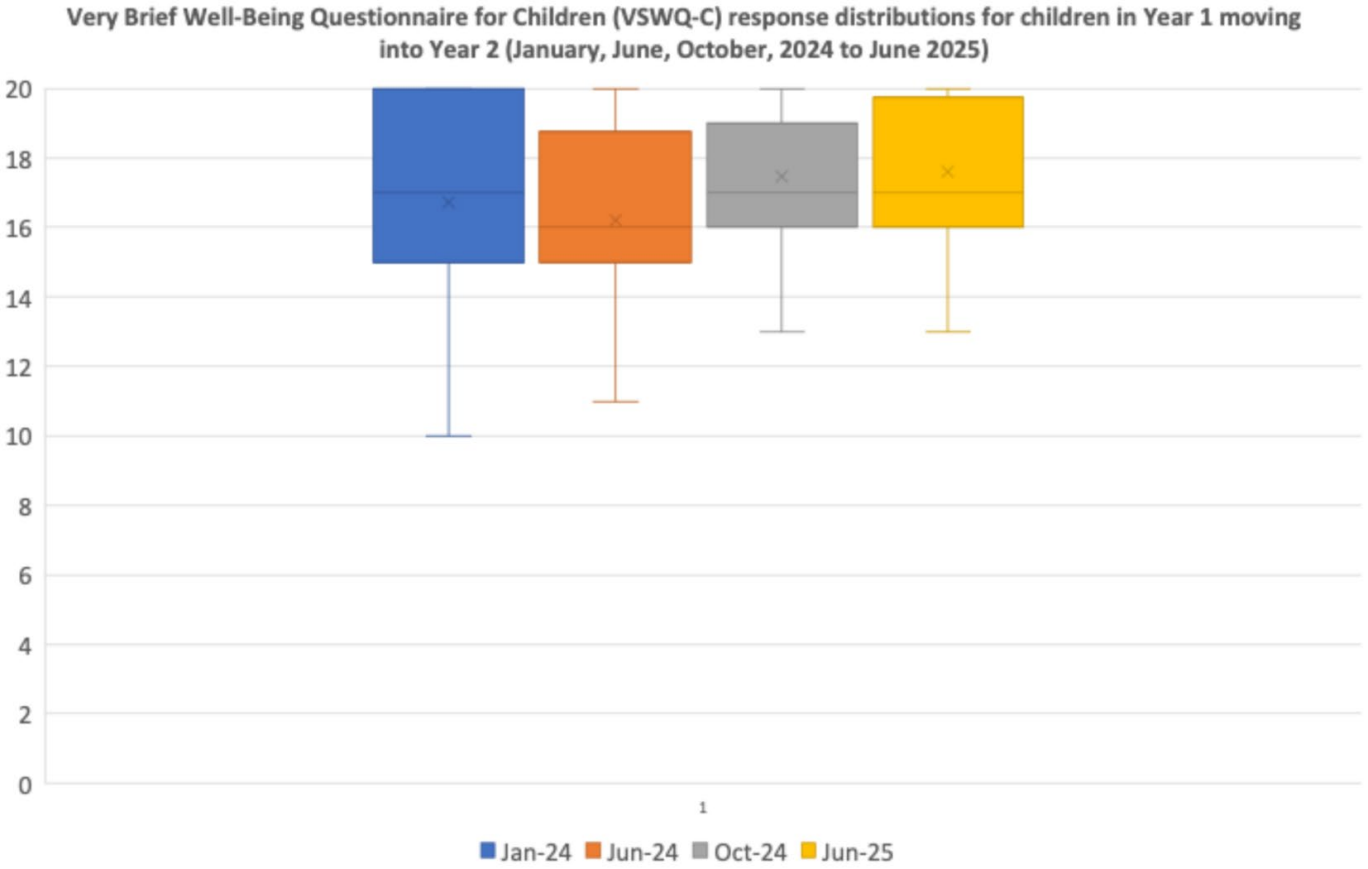
Very brief well-being questionnaire for children (VSWQ-C) response distributions for children in Year 1 moving into Year 2 (January, June, October 2024, and June 2025).

**Figure 5:**
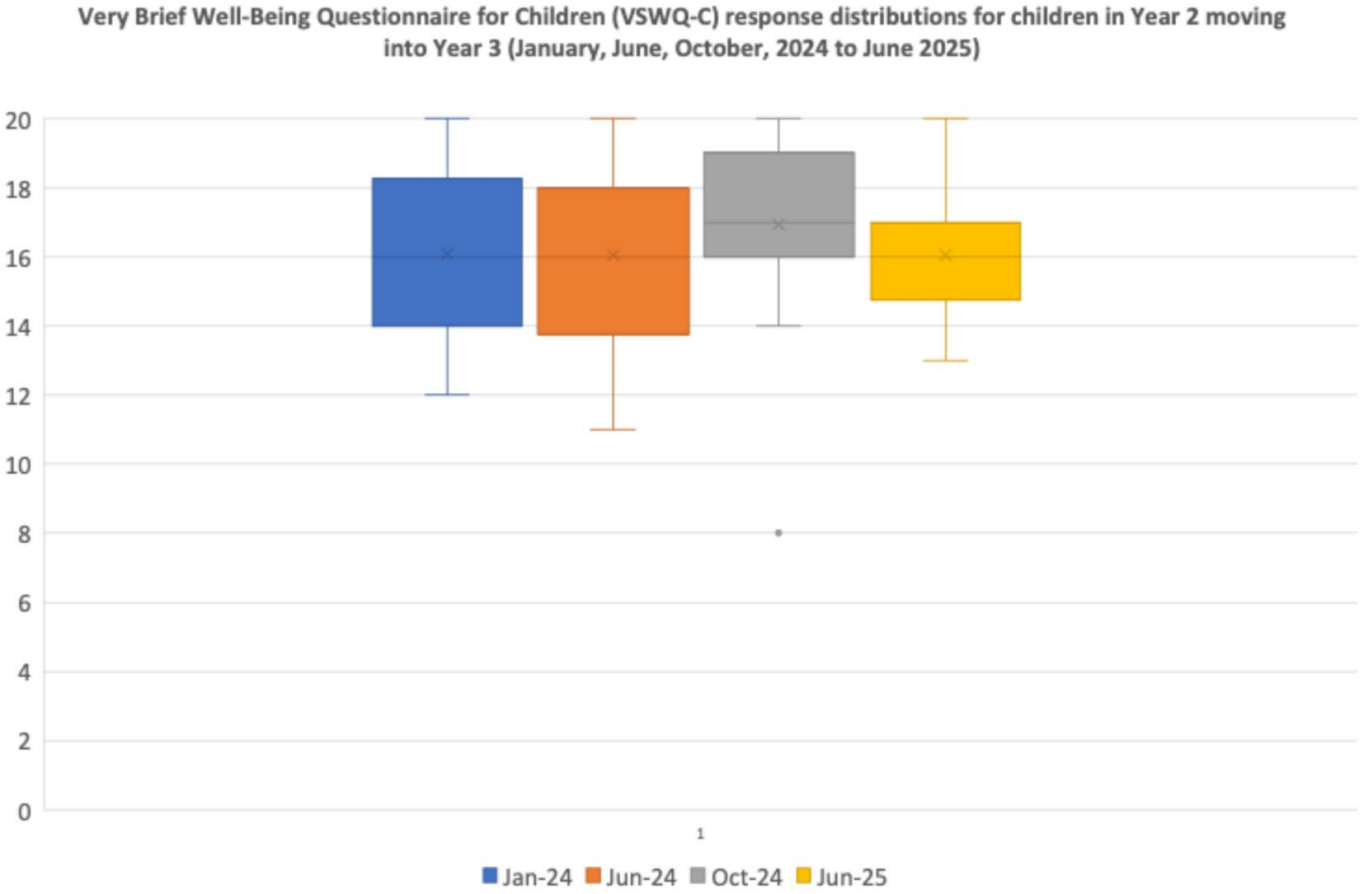
Very brief well-being questionnaire for children (VSWQ-C) response distributions for children in Year 2 moving into Year 3 (January, June, October 2024, and June 2025).

Children in both participant classes—Year 1 moving into Year 2, as well as Year 2 moving into Year 3—reported relatively high well-being ratings at each of the four assessment points, January (Baseline), June and October 2024, and June 2025 (see distributions of self-ratings in Figures 4, 5).

Overall, the self-reported well-being ratings on this well-being measure for both participant classes are positive. As the data were non-parametric, a Friedman Test for Repeated Measures was undertaken to compare the four assessment points. The data distributions in Figure 4 reflect a significant positive change for children’s ratings across Year 1 to Year 2 [*X2* (3) = 10.0, *p* < 0.02]; although these children were generally positive at baseline, their responses tended to become more closely clustered by June 2025.

In comparison, any changes in the well-being data for the older children moving across Year 2 to Year 3 were relatively small and non-significant statistically using the same Friedman Test for Repeated Measures [*X2* (3) = 2.51, *p* = 0.47, n.s.].

Furthermore, analysis of each individual response indicated that there is no evidence of any child reporting consistently less positive perceptions over time.

### Definitional positive and negative effect schedule for children

Data for the second of the two health and well-being measures, the *Definitional Positive and Negative Effect Schedule for Children (dPANAS-C)* derives from children’s responses to a 10-item self-report.^5^ The scale is split into two domains related to children’s feelings over the past week: *Positive Affect* relates to feelings such as happiness, pride, and activity, and *Negative Affect* relates to feelings such as sadness, fear, and anger (after Ebesutani et al., 2012). Children choose options in terms of their agreement with each individual item on a five-point scale, i.e., from ‘never/teeny bit’ to ‘very, very, very’—in line with Smees et al. (2020) adaptation of the original version by Ebesutani et al., 2012.

**Figure 6:**
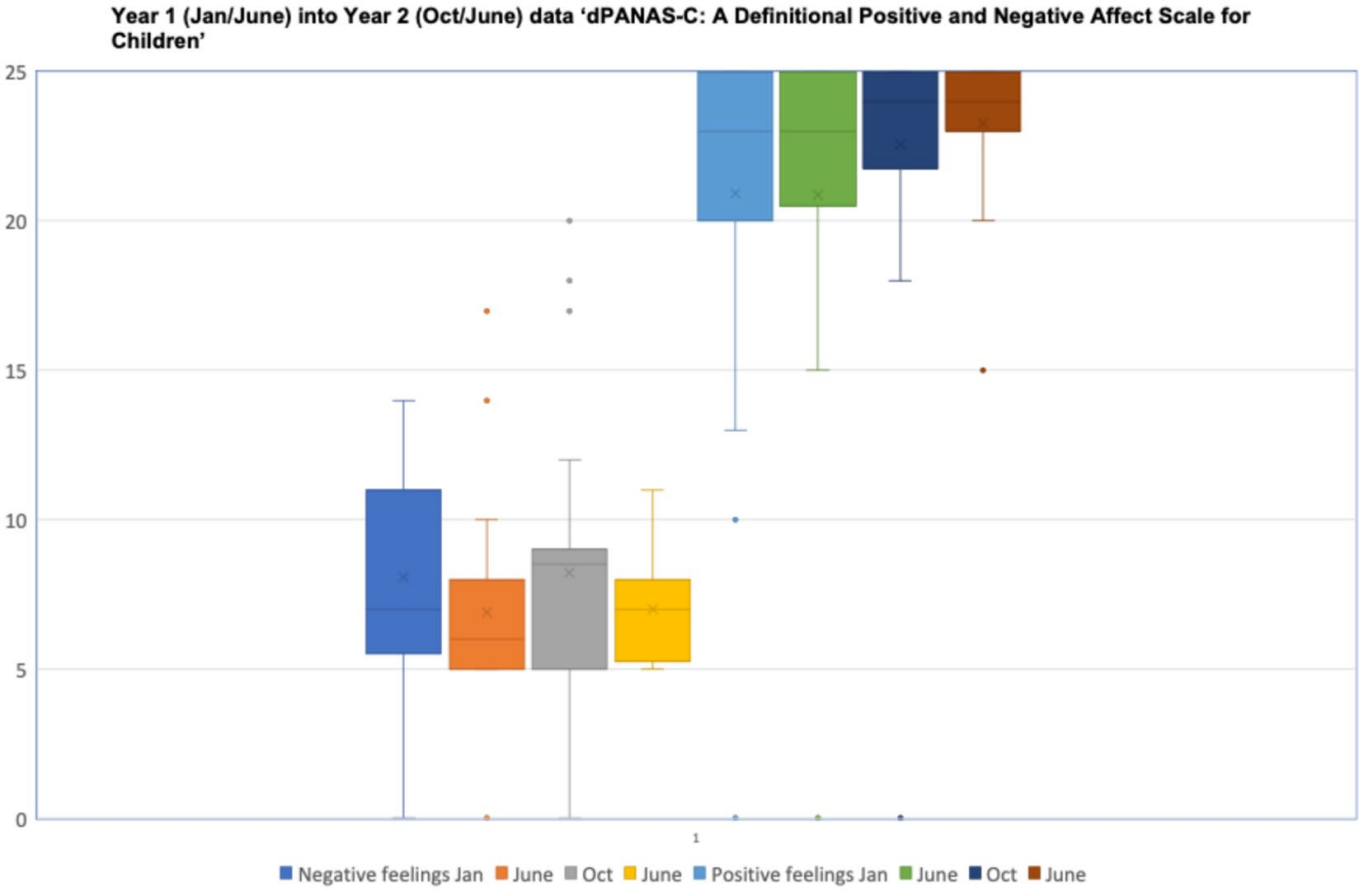
dPANAS-C: a definitional positive and negative affect scale for children*—*data distributions for children in year 1 moving into year 2 from January, June, October 2024 to June 2025.

Self-reported data from children in Year 1 moving into Year 2 using the *Definitional Positive and Negative Effect Schedule for Children (dPANAS-C)* revealed a consistent bias towards low negativity responses and high positive responses at each of the four assessment points (January, June and October 2024, and June 2025; Figure 6).

**Figure 7:**
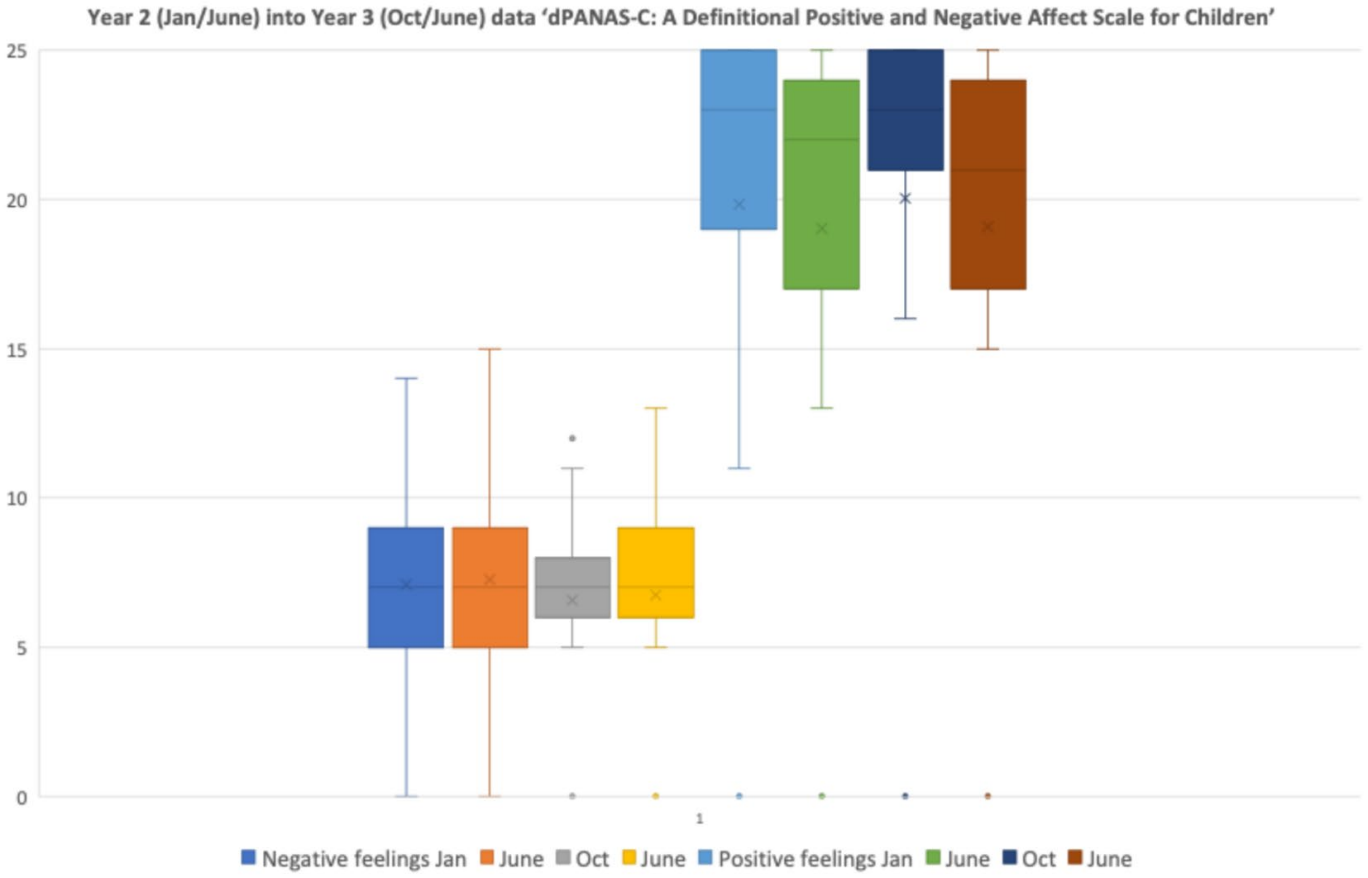
dPANAS-C: a definitional positive and negative affect scale for children—data distributions for children in Year 2 moving into Year 3 from January, June, October 2024, and June 2025.

A similar pattern of responses was evident for the children in the older class across Year 2 into Year 3 (Figure 7). Inspection of each child’s responses revealed that no individual pupil was consistent in reporting relatively high negative feelings, nor consistent in reporting relatively low positive feelings.

Although there was some variation in the distributions of self-reported *dPANAS-C* responses for children in each of the two focus classes, these were non-significant statistically, using Friedman’s Tests for Repeated Measures:

- Year 1–2: Negative: *X2*(3) = 3.36, *p* = 0.34, n.s.; Positive: *X2*(3) = 4.99, *p* = 0.17, n.s.
- Year 2–3: Negative: *X2*(3) = 0.21, *p* = 0.97, n.s.; Positive *X2*(3) = 2.45, *p* = 0.48, n.s.

Nevertheless, visual inspection of the data in Figure 6 implies that the younger children reduced the variability in their positive and negative responses over time, whereas the main variation for the older children was in reducing their negative responses (Figure 7). This variation appears to be cohort specific, in that the younger class had a different pattern of responses in the second year of data collection (aged 7+) as the older class in their previous year when they were also aged 7 + .

Given the evidence of possible ceiling effects in the health and well-being measures, with children being relatively positive at each assessment point, it was considered not to be appropriate to explore any statistical associations between these measures and those for singing. (Nevertheless, the research team separately decided to undertake a more nuanced picture of children’s singing experiences and singer identity via an exploratory ‘draw and tell’ study. This is under review for a separate publication.)

## Conclusion

Overall, the independent evaluation of data collected from participants in these two classes across these two school years (2024–2025) continues to provide evidence of the positive impact of the VOCES8 Foundation programme at least on children’s singing competency.

- Children’s singing competency continues to show a trend towards significant improvement over time.
- Collectively, the children are rated higher in their mean singing skills compared to *N* = 3,376 children of the same ages in our national dataset—data that were collected from 184 schools across the country as part of the *Sing Up* programme.
- The younger class children (moving from Year 1 to Year 2) made greater measured progress in their singing than the older children because, initially, their singing was comparatively less developed and so they had more opportunity to demonstrate improvement. In contrast, the older class of children (Year 2 to Year 3) had a higher mean singing competency initially and thus were already further forward in demonstrating their singing mastery—a common finding related to children’s age and singing behaviour in studies globally (e.g., Demorest and Pfordresher, 2015; Lu and Welch, 2025; Mang, 2006).
- Concerning any possible impact of singing on the children’s health and well-being, this is not evidenced statistically primarily because of the relative ceiling effects demonstrated in the data derived from two selected health and well-being measures. Across the evaluation period, children consistently rate themselves positively in their health and well-being, with some evidence of a trend towards even greater positivity in children from the younger class. Nor are there any individual children who might be identified from this dataset as particularly and consistently emotionally ‘vulnerable’.
- One inference is that the VOCES8 singing development programme is making an important contribution to the life and culture of the children and their school, and this may provide a preventative and protective measure for children’s health and well-being, enabling them to be more resilient to the challenges that they face. However, this is speculative.
- This supposition is based on the background literature rehearsed at the beginning of this report which offers a diverse range of international evidence concerning the potential wider benefits of successful singing, both individual and collective, across the lifespan. Thus, it is possible to speculate that the persistence of positive self-ratings by these children may relate to the beneficial impacts of their singing experiences, both in school and elsewhere.
- Indeed, our separate report on the older children’s identities as singers (Baxter & Welch, in review), elicited by a draw-and-tell method, confirms that they are positive about singing and the experiences of singing in their lives, both in and out of school.
- Also, the finding that these children are sustaining a positive identity as singers contrasts somewhat with our national dataset finding that, paradoxically, children tend to like singing in school less and less as they get older, despite becoming more skilled (e.g., see also De Vries, 2010; Hargreaves et al., 2015). ‘School music’ can become perceived as a separate category of music which is less appreciated than music outside school, although the evidence is mixed over time (Harland et al., 2000; Lamont et al., 2003; Bath et al., 2020; Newton et al., 2025). However, a caveat here is that, if children experience a very successful singing culture, this negativity towards school singing is likely to be either much less evidenced or non-existent.

There are several limitations in the design of the study which need to be acknowledged. In this double-group longitudinal comparison, whereas the singing data could be contextualised against a national dataset, it was not possible to include an external matched control group for the health and well-being measures. The children were familiar with the research team and the protocol, and care was taken to ensure that each participant reflected on their answers to the tablet-based Likert items. Nevertheless, it may be that some of the children found the statements somewhat abstract. It would be useful to have a larger sample of participants undertake these health and well-being measures to gain insight into the possible range of variables that might impact responses. It should also be recognised that the singing programme is being provided by a specialist expert team of professional singers which may underlie the significant changes reported in singing assessment data. Again, it would be useful to investigate the impact of regular singing in the context of a less specialised intervention.

Nevertheless, within the constraints of this particular research context, the emergent data are useful in understanding the longitudinal changes in focused measures for these two school classes and which offer insights related to singing behaviour and development. It has been argued elsewhere that successfully engaging in collective musical activity is an embodied expressive interaction and thus can promote resilience (Nijs and Nicolaou, 2021). While there is no direct evidence in this particular case, there is an implication that regular positive group singing experience has the potential to be protective and supportive of health and well-being.

## Data Availability

The raw data supporting the conclusions of this article will be made available by the authors, without undue reservation.

## Footnotes

1 These and related studies are reviewed in a new Routledge book chapter on singing, well-being and social inclusion, see Welch, in press, ‘Singing, wellbeing and social inclusion in children: a multimodal experience’ (expected 2026).

2
https://voces8.foundation/voces8-method

3 For more detail on the background to this research evaluation, see the report by Welch and Baxter (2025) on the first year of the VOCES8 Foundation programme at the school.

4
https://www.singandsee.com

5 The *dPANAS-C* scale was as adapted by Smees et al. (2020) with permission from the original by Ebesutani et al. (2012), which was itself an adaptation of earlier scales with more items by Watson et al. (1988) and Laurent et al. (1999).
